# Gemcitabine Peptide-Based Conjugates and Their Application in Targeted Tumor Therapy

**DOI:** 10.3390/molecules26020364

**Published:** 2021-01-12

**Authors:** Aleksandra Hawryłkiewicz, Natalia Ptaszyńska

**Affiliations:** Department of Molecular Biochemistry, Faculty of Chemistry, University of Gdańsk, Wita Stwosza 63, 80-308 Gdańsk, Poland; aleksandra.hawrylkiewicz@phdstud.ug.edu.pl

**Keywords:** gemcitabine, peptide-drug conjugates, targeted drug delivery, anticancer therapy

## Abstract

A major obstacle in tumor treatment is associated with the poor penetration of a therapeutic agent into the tumor tissue and with their adverse influence on healthy cells, which limits the dose of drug that can be safely administered to cancer patients. Gemcitabine is an anticancer drug used to treat a wide range of solid tumors and is a first-line treatment for pancreatic cancer. The effect of gemcitabine is significantly weakened by its rapid plasma degradation. In addition, the systemic toxicity and drug resistance significantly reduce its chemotherapeutic efficacy. Up to now, many approaches have been made to improve the therapeutic index of gemcitabine. One of the recently developed approaches to improve conventional chemotherapy is based on the direct targeting of chemotherapeutics to cancer cells using the drug-peptide conjugates. In this work, we summarize recently published gemcitabine peptide-based conjugates and their efficacy in anticancer therapy.

## 1. Introduction

Improving the therapeutic index of anticancer agents is an enormous challenge. In a time when the number of patients suffering from a cancer-related disease has been increasing each day and when conventional therapies gather a worrying number of deficits and drawbacks [1,2], new treatment options are required to relieve the symptoms and ultimately to eradicate the disease. Over the past decades, the development of new therapies that are more selective and less harmful to patients has been the target of many research groups [3,4]. However, these therapies still carry a risk of relapse and numerous side effects. The chemotherapeutic agents that target rapidly dividing cancer cells significantly damage healthy cells, especially those with rapid growth, such as bone marrow, gastrointestinal mucosa, and hair cells [5,6]. This causes the most common side effects of chemotherapy, such as myelosuppression (reduced blood cell production), inflammation of the lining of the gastrointestinal tract, and hair loss [6].

A modern approach to improve conventional chemotherapy is to focus on the direct targeting of chemotherapeutic agents to cancer cells [3,5]. Targeted drug delivery methods have been developed to improve drug efficacy and lower side effects by directing the drug to a specific cell type, enhance the tumoricidal effect, and reduce the peripheral toxicity of a specific drug [7]. Targeting decreases the side effects of therapeutic agents by delivering drugs to the intended destination [8]. To optimize the strategy of the anti-cancer agents targeting, one must ensure that the drug will not affect non-tumor-transformed cells and that the active substances will be transported in sufficient amounts to eliminate cancer cells and inhibit the tumor growth [8,9].

Designing the carriers of therapeutic substances is an approach that enables not only the improvement of pharmacokinetic properties and the biodistribution of traditional and innovative drugs, but also a reduction of their numerous side effects [9]. The binding of a drug to a carrier is often accompanied by a change in its mode of delivery, which is advantageous if it leads to the increased accumulation of the drug in the target tissue, e.g., in a cancer cell [5,8]. The biodistribution of such a conjugate is highly dependent on the properties of the carrier [10]. The synthesis of drug–peptide conjugates for the targeted delivery to a specific group of cells usually involves the conjugation of the drug with a targeting peptide via an appropriate linker, which could in turn facilitate the chemical or enzymatic release of the drug once the conjugate enters cancer cells via a receptor–mediated endocytosis mechanism [8,11]. The ester linkages, among several functional groups employed to connect the drug to the linker, have been widely utilized due to its possible release via an enzymatic (i.e., esterase–based) hydrolysis of the ester bond [12].

Several peptide drug conjugates have been developed for different cancer types [13,14,15,16,17]. Some of these endeavors proceeded to clinical rating [16,17], while other provided crucial information about approaches that could enhance the stability of these molecules in circulation, thus improving their efficacy and reducing any associated toxicity [18].

**Figure 1:**
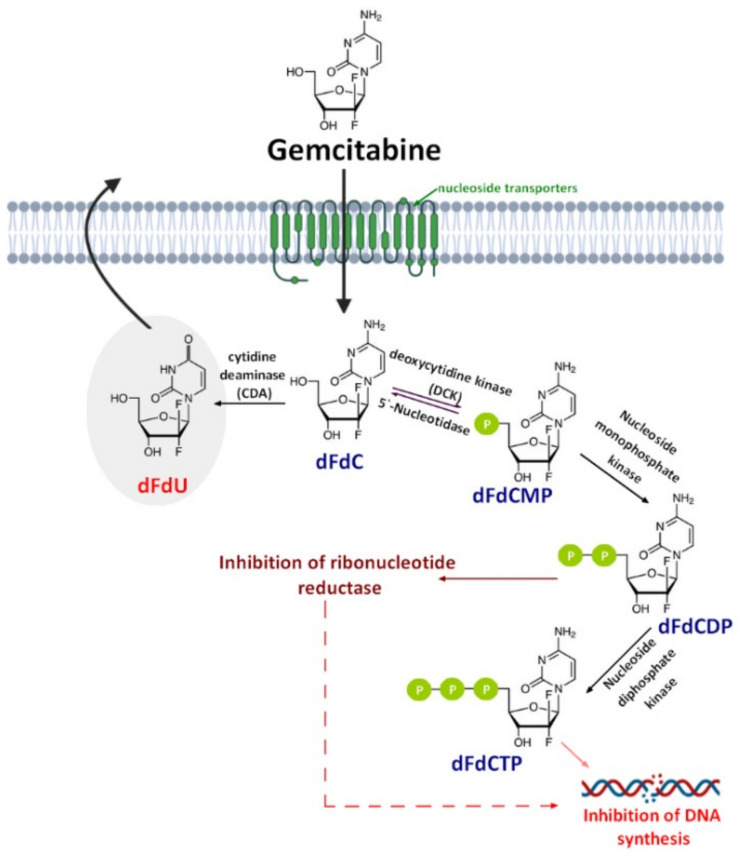
A schematic of gemcitabine (dFdC) cellular uptake, mechanism of action, and metabolism. dFdCMP: Gemcitabine monophosphate; dFdCDP: Gemcitabine diphosphate; dFdCTP: Gemcitabine triphosphate; and dFdU: 2′,2′-difluoro-2′-deoxyuridine.

Gemcitabine (2′,2′-difluorodeoxycytidine) (dFdC, Gem-used in the conjugate’s names)—a pyrimidine antimetabolite—is a prodrug with a demonstrated efficacy against a wide variety of cancers [19,20,21] and has been approved for use against colon, non-small cell lung, breast, pancreatic, bladder, and ovarian cancers [22,23]. Gemcitabine is transported into cells by nucleoside transporters: Human equilibrative (hENT1, hENT2) and concentrative nucleoside transporters (hCNT, hCNT2, and hCNT3) [24]. The drug molecule is then activated by deoxycytidine kinase (DCK)—a rate-limiting step for its pharmacological action—and phosphorylated to difluorodeoxycytidinemonophosphate (dFdCMP), which is further phosphorylated by the phosphate kinase enzyme into diphosphate (dFdCDP) and triphosphate (dFdCTP) forms. Both the dFdCDP and dFdCTP active forms show antitumor activity by the inhibition of the cellular DNA synthesis. dFdCTP incorporates into the DNA leading strand inhibiting DNA synthesis and subsequently leading to cell apoptosis. dFdCDP also has an indirect cytotoxic effect caused by the inhibition of ribonucleotide reductase. Finally, dFdC is rapidly metabolized into its inactive metabolite—2′,2′-difluoro-2′-deoxyuridine (dFdU)—by cytidine deaminase present in the blood, liver, and kidneys and is excreted through urine [25] (Figure 1).

Despite the clinical successful application of gemcitabine, its short plasma half-life (9−13 min in human plasma) [26], poor diffusion into cells, and adverse toxicity—such as myelosuppression, the principal dose-limiting toxicities in gemcitabine cancer therapy—significantly reduce its chemotherapeutic potential [27,28]. This short half-life is the result of deamination of gemcitabine by cytidine deaminase which—as mentioned before—metabolize gemcitabine to the inactive dFdU [29]. Likewise, phosphorylated metabolites of gemcitabine are inactivated via the reduction by cellular 5′-nucleotidase (5′-NT) and then are rapidly removed from the body by the enzymatic conversion of gemcitabine [30,31]. Another important drawback associated with gemcitabine therapy is the drug resistance related to the nucleoside transporter deficiency, which is developed by some tumor cells after the initial tumor regression [32]. For this reason, many approaches have been made to improve the safety profile of gemcitabine and increase its chemotherapeutic index. These approaches include both chemical modifications either on the cytosine’s aniline or on the 5′-hydroxyl group of the 2,2′-difluoro-2′-deoxyribose moiety [33] and the novel drug delivery technology. Until now, various delivery strategies such as liposomes [34,35,36], nanoparticles [37,38], lipidic and nonlipidic derivatives [36], as well as poly(ethylene glycol) (PEG) and other polymeric drug conjugates [39,40] have been studied to prevent rapid plasma degradation and improve the selective delivery of gemcitabine to the tumor tissue. These approaches have been widely discussed earlier [25] and will not be discussed again herein.

An alternative strategy, which has recently attracted much more attention, was established by chemically conjugating hydrophilic drugs to cell-penetrating peptides (CPPs) [41,42]. CPPs are relatively short peptides, typically composed of less than 30 amino acid, that have been shown to be comparatively non-cytotoxic and capable of crossing the cell membrane. These peptides have been used to facilitate the transport of various therapeutic agents into cells, including plasmid DNA, siRNA, therapeutic proteins, viruses, imaging agents, and other various nanoparticles. The coupling of the anticancer drug to CPPs may result in numerous advantages, such as improved solubility, intracellular uptake, biodistribution, and pharmacokinetic profiles. Therefore, the CPP-based drug delivery system offers great potential for improving the intracellular delivery of therapeutic agents with poor permeability [42].

In this work, we review recently published in vitro and in vivo studies on gemcitabine peptide-based conjugates, especially with cell-penetrating peptides (CPPs) and receptor-binding peptides, and their efficacy in anticancer therapy. In Table 1 we summarize all the peptide conjugates with gemcitabine described until today and their effectiveness in the tested cell lines.

## 2. Gemcitabine Conjugated with Cell-Penetrating Peptides (CPPs)

In recent years, numerous natural and synthetic CPPs—such as TAT, transportan, penetratin peptides, and polyamino acids (e.g., poly-arginines)—were utilized for the intracellular delivery of anticancer agents [51,52]. Since all CPPs are able to efficiently pass through cell membranes while being non-cytotoxic and carry a wide variety of cargos inside cells, they are also used to form conjugates with gemcitabine.

**Figure 2:**
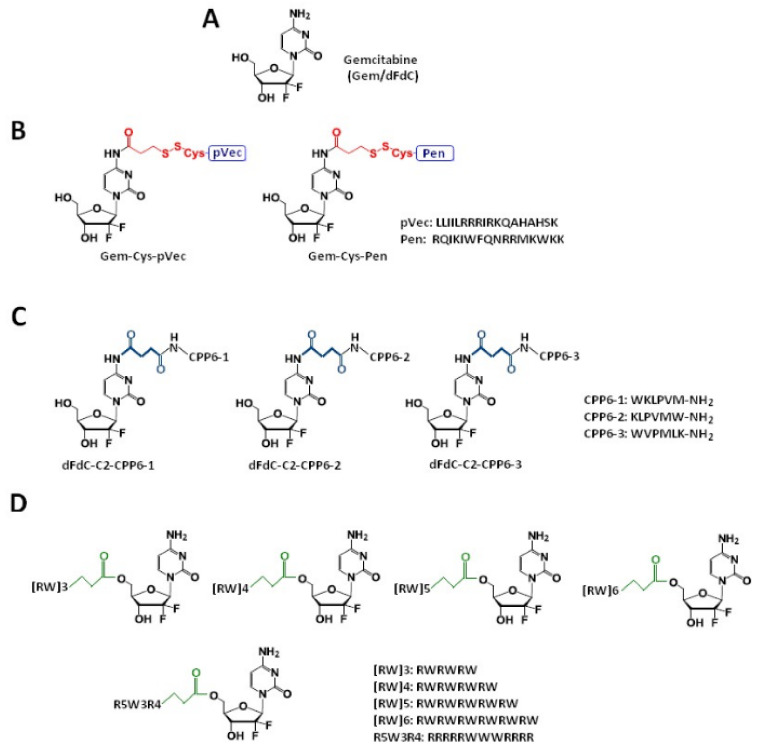
Chemical structures of gemcitabine (**A**) and their conjugates with CPPs and their constituents. (**B**) Gem-Cys-Pen and Gem-Cys-pVec conjugates: Conjugates of gemcitabine with Penetratin and pVec; (**C**) CPP6–dFdC: Conjugates of gemcitabine with CPP6; and (**D**) conjugates of gemcitabine with arginine and tryptophan-rich CPPs.

Vale et al. [41] synthesized two novel peptide–gemcitabine conjugates using two well-known CPP sequences—Penetratin (Pen, RQIKIWFQNRRMKWKK) [53] and pVEC (LLIILRRRIRKQAHAHSK) [54]. The authors connected these peptides with the aniline moiety of gemcitabine through the 3-sulfanylpropanoyl linker. They added an additional cysteine residue to the N-terminus of both CPPs to couple them with the linker, receiving Cys-Pen and Cys-pVec. A disulfide exchange reaction between Cys-modified CPPs and gemcitabine connected with the linker produced target conjugates (Figure 2B). The time-dependent kinetics of the gemcitabine release from hydrolysis of these new conjugates was studied in phosphate-buffered saline (PBS) at pH 7.4, 37 °C, and their biological activity was evaluated using three human cancer cell lines—MKN-28 (human gastric cancer), Caco-2 (heterogeneous human epithelial colorectal adenocarcinoma), and HT-29 (human colon adenocarcinoma). The results showed an increase in the anti-proliferative activity of gemcitabine in vitro upon conjugation with the CPP. Both CPP–gemcitabine conjugates (with the only exception of the Gem-Cys-pVEC conjugate against Caco-2 cells) worked substantially better than their components alone (either as a free drug or Cys-CPP) on the tested cell line (IC_50_ < 50 µM for conjugates vs. IC_50_ > 100 µM for gemcitabine and Cys-CPP). A semi-quantitative study of the degradation kinetics of both conjugates in PBS at pH 7.4, 37 °C, showed that gemcitabine is released upon the hydrolytic cleavage of the aromatic amide in Gem-Cys-Pen and Gem-Cys-pVec conjugates, with half-lives of approximately 9.6 days and 42 h, respectively. Based on the results mentioned above, the Vale’s group suggests that the remarkably higher stability of this conjugate may underlie its ability to make full use of its CPP moiety for enhanced internalization into the target cells, with a more controlled release of the parent drug over time [41].

In order to study the pharmacokinetics of gemcitabine-CPP conjugates and its constituents (gemcitabine and respective CPPs) and to establish a possible relation between the penetration potency of CPP and their physicochemical properties, Ferreira et al. [55] used the computational tool GastroPlus™—a powerful mechanistically-based simulation and modeling software for pharmaceutical research. Based on the simulations carried out in GastroPlus™, the authors stated that the conjugates’ bioavailability is ensured and the plasma concentration should reach therapeutic levels. The calculated AUC (area under the plasma concentration–time curve, μg-h/mL) for the conjugates was comparable to the AUC calculated for gemcitabine (~7.4404 and 7.4368, respectively). Yet, the estimated C_max_ (maximum plasma concentration, in μg/mL) was higher for all the peptides and the analyzed conjugates (C_max_ = 7.4403 μg/mL) compared with gemcitabine alone (C_max_ = 5.9505 μg/mL). The Gem-Cys-pVEC conjugate binds less extensively to plasma proteins (>F_up_, 42.89%). Bearing in mind that this conjugate showed the best in vitro bioactivity result for MKN-28 and HT-29 cells (IC_50_ = 20.68 µM and 45.20 µM, respectively; IC_50_ > 100 µM for gemcitabine) and released gemcitabine in PBS faster than the Gem-Cys-Pen conjugate (50% over 42 h versus 9.6 days for Gem-Cys-Pen) [41], the authors suggested that Gem-Cys-pVec conjugate has the most suitable profile for the drug delivery [55].

Continuing the previous studies, Vale’s group synthesized new series of gemcitabine-CPPs conjugates [32] containing three novel hexapeptides—CPP6-1, CPP6-2, and CPP6-3—which are the analogues of two peptides—KLPVM and VPMLK—derived from a family of CPP5 [56]. These peptides were reported to have a high ability to cross cell membranes. To improve their cell penetration capacity, additional tryptophan residues at their N- or/and C-termini were added and linked with gemcitabine using succinic anhydride, resulting in three novel gemcitabine-CPP6 conjugates (Figure 2C). Such a linker should improve the rate of drug delivery, as it protects the drug from cytidine deaminase (CDA) due to the conversion of its amino group to an amide moiety, comparatively unreactive under physiological conditions. To evaluate the in vitro cytotoxicity of the synthesized conjugates, the authors used human pancreatic adenocarcinoma (BxPC-3), human breast adenocarcinoma (MCF-7), and human prostate adenocarcinoma (PC-3) cancer cell lines. The results showed that two of three synthesized conjugates (dFdC-C2-CPP6-1 and dFdC-C2-CPP6-3) displayed a significantly higher cell growth inhibitory activity against PC-3 cells as compared with gemcitabine and CPPs constituent (after the conjugation with CPP6-1 and CPP6-3, gemcitabine IC_50_ decreased from 74 nM to 15 nM and 14 nM, respectively). Moreover, the three new conjugates of gemcitabine with CPP6 presented more potent cell growth inhibitory activity in MCF-7 and PC-3 cells (IC_50_ < 7 nM and IC_50_ ≤ 15 nM, respectively) than the reference drugs, tamoxifen (IC_50_ = 20 nM for MCF-7 and IC_50_ > 1000 nM for PC-3 cells) or metformin (IC_50_ = 9.9 nM for MCF-7 and IC_50_ = 189 nM for PC-3 cells) with the exception of the dFdC-C2-CPP6-2 conjugate for the PC-3 cell line (IC_50_ > 1000 nM). In addition, during this study the authors confirmed that in BxPC3 cells the dFdC-CPP6 conjugates are transported preferentially by hENT-1 transporter, and that once in the cytoplasm, dFdC-CPP6 conjugates may undergo sequential phosphorylations, disrupting DNA synthesis and causing apoptosis [32].

### 2.1. Gemcitabine Conjugates with Arginine-Rich CPPs Modified at Tryptophan Residues

Arginine-rich cell-penetrating peptides are short cationic peptides that are able to traverse plasma cell membranes. Although the effective delivery of many biologically active macromolecules into cells has been achieved using these peptides, their mechanisms of membrane crossing are still unknown. Electrostatic interactions are important for arginine-rich cell-penetrating peptides, but non-electrostatic effects such as hydrophobicity and peptide structural transitions can also contribute to the binding affinity of amphipathic cell-penetrating peptides to cell membranes [57,58]. Previous studies show that the presence of l-tryptophan residues enhances the penetrating properties of arginine-rich CPPs [58]. As a result of its aromaticity, l-tryptophan has a strong preference for interactions with the cell bilayer facilitating the CCPs anchoring to membrane proteins [59,60,61,62]. Not only the number but also the character of hydrophobic residues, their position, and interfacial properties of CPPs are important for the membrane crossing. Penetrating abilities are favored when the tryptophan residues form a large hydrophobic peptide layer.

Zakeri-Milani et al. examined in their work [42] the effect of the gemcitabine coupling with arginine and tryptophan-rich CPPs on the drug toxicity in A549 cell line. They synthesized the conjugates where gemcitabine was covalently attached to the five peptides (named as R5W3R4, [RW]6, [RW]3, [RW]4, and [RW]5, Figure 2D) using the succinyl hydrolysable spacer enabling the drug release (by esterases) after uptake into the cells [63]. The results showed that three of five peptides improved the cytotoxicity of gemcitabine. The coupling of arginine-tryptophan block CPPs, such as R5W3R4, [RW]6, and [RW]3, with gemcitabine leads to the increased toxicity compared with the free drug. The CPPs conjugated with gemcitabine did not exhibit increased cytotoxicity at concentrations less than 10 µM in comparison to the free drug. Nevertheless, Gem-R5W3R4, Gem-[RW]6, and Gem-[RW]3 conjugates showed reduced cell viability at 15 and 25 µM. Gemcitabine revealed 20% cell activity at concentrations of 15 and 25 µM. The value of cell growth was reduced to 16% and 6% for Gem-R5W3R4 at 15 and 25 µM, respectively. In the case of Gem-[RW]6, the cell viability decreased to 14% at 15 and 25 µM. Among the five peptide-drug conjugates, Gem-[RW]3 displayed the highest cytotoxicity at 15 and 25 µM—the cell viability decreased to 9% and 5%, respectively [42]. In this study, the authors also confirmed that this class of CPPs improves the intracellular delivery of the drug into tumor cells as well as its activity. As mentioned above, the cellular uptake of gemcitabine is mostly mediated by hENT1 transporters. Cells with the decreased expression of hENT1 exhibit a lower susceptibility to gemcitabine toxicity by a blockade of the drug cellular uptake [64]. Cell-penetrating peptides transport cargo molecules across the plasma membrane by the direct penetration or endocytosis, bypassing the transporter mediation. Thanks to that, the gemcitabine resistance connected with hENT1 expression disorders can be reduced using the CPPs conjugates.

### 2.2. Gemcitabine Conjugated with Receptor-Binding Peptides

**Figure 3:**
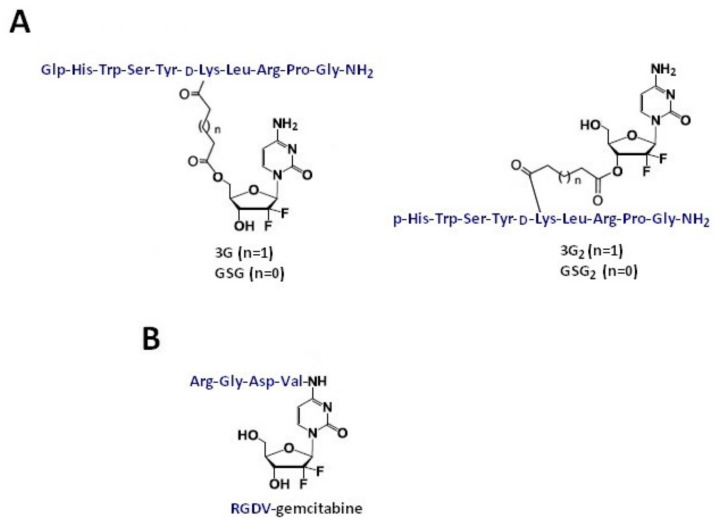
Chemical structures of gemcitabine conjugates with receptor-binding peptides. (**A**) Conjugates of gemcitabine with GnRH-R ligand peptide and (**B**) conjugate of gemcitabine with RGDV peptide.

Another recently developed approach to tackle challenging cancer types is based on connecting the chemotherapeutic agents with the peptide ligands which show a high affinity for receptors overexpressed in a tumor tissue, such as the gonadotropin releasing hormone receptor (GnRH-R). Karampelas et al. [43] developed gemcitabine conjugates. In this approach, gemcitabine conjugated to the GnRH-R ligand peptide ([D-Lys^6^]-GnRH) by the ester linkages (applying four or five carbons, glutaric or succinic anhydride) at the primary 5′-OH or secondary 3′-OH group of the drug, obtaining gemcitabine-succinate-GnRH conjugates (Figure 3A). NMR studies using 2D NMR TOCSY experimentation showed that the conjugation of gemcitabine with GnRH peptide does not significantly change the structural conformation of the peptide required for a successful binding to the GnRH-R. Moreover, the presented GnRH−gemcitabine conjugates exhibited high binding affinity for the GnRH-R-, suggesting that these types of molecules can support the GnRH-R targeted delivery strategy. In this work, GnRH-gemcitabine conjugates were assessed for their antiproliferative activity against two androgen-independent CaP cell lines (DU145 and PC3) and were ultimately subjected to pharmacokinetic evaluation in mice. The results showed that three of the synthesized conjugates—namely, 3G_2_, GSG, and GSG_2_—display high antiproliferative activity in CaP cell lines tested in comparison with gemcitabine (average IC_50_ values for 3G_2_, GSG, and GSG_2_ of 663, 308, and 439 nM, respectively, versus 231 nM for gemcitabine). Gemcitabine-succinate-GnRH (GSG) was selected as the lead candidate compound among GnRH gemcitabine conjugates for the efficacy studies in mice. This selection was based on its high antiproliferative in vitro activity, along with significant metabolic stability and pharmacokinetic advantages, and a slower inactivation of gemcitabine in comparison with gemcitabine. The administration of GSG in tumor-bearing NOD-SCID mice showed that GSG has a tumor bioavailability, as it can be delivered at the tumor site at appreciable levels even at a lower dose in comparison with the efficacious dose. Finally, the treatment of GnRH-R-positive xenografted mice with GSG showed a significant advantage in tumor growth inhibition when compared with the control group (vehicle) or the equimolar doses of gemcitabine or [D-Lys^6^]-GnRH. The average tumor volume of the GSG-treated group at day 18 was 506 mm^3^ ± 152, significantly lower (*p* < 0.001) when compared with the vehicle, low dose gemcitabine, or [D-Lys^6^]-GnRH treatments. Due to the fact that GSG efficacy was achieved with a significantly lower dose when compared with gemcitabine, Karampelas et al. suggested that the GSG prodrug could ultimately lead to a significant reduction of the gemcitabine’s effective dose and adverse effects [43].

#### Gemcitabine-Succinate-GnRH Conjugates with Improved Metabolic Properties and Dual Mode of Efficacy

Karampelas et al. in the next work [18], performed a series of studies aiming to analyze the molecular pathways involved in the GSG’s mechanism of action. The cell uptake study showed that the GSG conjugate can shift the dynamic balance of active (formation of dFdCTP) vs. inactive (formation of dFdU) metabolites towards the advantageous side affording a higher anticancer efficacy. Moreover, they confirmed that the GSG conjugate can release gemcitabine intracellularly [18], a process mediated by nucleoside transporters. This finding opens the opportunity to develop new molecules that could pass through membranes, such as the lysosomal membrane. Based on this phenomenon, one could avoid nucleoside transporter mediation, since the nucleoside transporter deficiency is one of the most common forms of gemcitabine-associated resistance [24,33]. Karampelas et al. demonstrated that GSG present less hematotoxic potential compared with gemcitabine (IC_50_ value 24.3 ± 6.4 nM for GSG vs. 12.1 ± 6.7 nM for gemcitabine). These results suggest that GSG could be less hematotoxic compared with gemcitabine, which is potentially valuable from a clinical perspective. In addition, the authors observed no hematological or other toxicities after a daily repeated dosing protocol of GSG in mice, unlike for gemcitabine which causes hematological toxicities. These important findings could be a stepping stone towards the reevaluation of gemcitabine being a part of a peptide-drug conjugate as a therapeutic option for prostate cancer [18].

The results discussed above support the advantages of using cell-penetrating peptides for the improvement of gemcitabine intracellular delivery in a tumor as well as its cytotoxic activity. The majority of the presented conjugates presented more potent cell growth inhibitory activity, especially in MCF-7 and PC-3 cells, than the reference drugs, tamoxifen or metformin. Additionally, these conjugates displayed increased toxicity compared to free gemcitabine and improved gemcitabine metabolic stability and have the potential to limit drug resistance. Moreover, the conjugation of gemcitabine with CPPs enables the enhancement of the anti-proliferative performance of that parent drug, which is beneficial for anticancer therapies. To sum up, gemcitabine conjugated with CPPs can be considered an interesting approach to developing therapeutic agents for prostate cancer treatment.

## 3. RGD Peptides-Gemcitabine Conjugates

Synthetic peptides containing the RGD sequence constitute one of the major classes of new pharmaceuticals with integrins as their primary therapeutic target. Such peptides act by binding to integrin receptors and destroying the mitochondria after cell penetration. This affects angiogenesis, thus disrupting the process of creating new blood vessels and inhibiting the tumor growth.

Unmodified RGD-peptide (Arg-Gly-Asp) binds specifically to αVβ3 and αVβ5 receptors, such as fibrinogen, fibronectin, vitronectin, plasminogen, thrombospondin, prothrombin, MMP-2, laminin, osteopontin, etc. which are excessively expressed on tumor cells and surfaces of vasculature, and is applied as an important component of a delivery system of various agents: Anticancer drugs, nanoparticles, imaging compounds, and virus vectors to tumor or angiogenic vessels [65,66,67,68,69]. The affinity of RGD peptides for their molecular targets may be affected by their conformation [70,71]. Apart from the direct interactions between peptide’s functional groups and their receptors, the conformational properties of the RGD motif can also be modified. The cyclization is a common modification, improving the binding features of RGD peptides and the rigidity of the molecule. Linear RGD peptides are strongly susceptible to the enzymatic degradation, but the introduction of conformational constrains caused by the cyclization averts this process, so cyclic peptides are more stable, more potent, and more specific. In cyclic peptides, the RGD-peptide sequence is surrounded by other amino acid residues to build a ring system. These modification offers the possibility to present the RGD sequence in a specific conformation for a selected integrin [70,71].

### 3.1. Co-Administration of iRGD Peptide and Gemcitabine

The iRGD (C(&)RGDKGPDC(&)) is one of the most commonly used tumor-targeting peptides. This peptide binds to the αVβ3 and αVβ5 integrins and to neuropilin-1 (NRP1) receptors increasing the pore diameter and surface area of blood vessels in the tumor, reducing the influence of pressure in its interstitium, and, in consequence, increasing the diffusion rate of small molecule drugs [69]. Drugs, nanoparticles, or proteins conjugated with iRGD can be directly administered to the tumor parenchyma, which reduce the side effects of the chemotherapy. Due to this innovative delivery system and the low toxicity to normal cells, iRGD has drawn the attention of several research groups [67]. Co-administration of this peptide has been proven to significantly enhance the intratumoral accumulation of various molecules, such as, doxorubicin (7-fold), dextran (3- to 5-fold), Evans blue dye (2- to 4-fold), trastuzumab (40-fold), doxorubicin-liposome (14-fold), and nab-paclitaxel (9- to 12-fold) in the mouse breast or prostate cancer models [72].

Akashi et al. [44] demonstrated that the co-administration of gemcitabine and iRGD peptide boost the anticancer effect of the therapy. Studies conducted on the five mouse pancreatic cancer cell xenograft models showed that the high expression of neuropilin-1 enhanced the anticancer effect of gemcitabine combined with iRGD in comparison with its monotherapy. They also proved that there is a significant difference in the overall survival between patients showing high NRP1 expression (0.5–46.6 months) and low NRP1 expression (5.6–94.6 months), which implies that co-administration of the toxic agent with iRGD peptide could be beneficial for cancer patients with NRP1-overexpressing tumors.

### 3.2. RGD Peptide-Gemcitabine-Loaded Nanocarriers

RGD-targeted nanocarriers are favorable in delivering chemotherapeutics, peptides, and proteins or nucleic acids. Nanocarriers, such as nanoparticles, liposomes, micelles, and others, can be loaded at their surface with a targeting ligand such as an RGD-based peptide. This kind of targeted therapy offers many benefits. Firstly, the size of nanocarriers (from 20 to 400 nm) activates the passive targeting process, which increases the permeability and retention drug effect (EPR) [73]. Secondly, the size of nanocarriers enable the evasion of the renal filtration causing the prolonged clearance and, finally, the longer accessibility of the ligand to the target receptors in a tissue [74]. Thirdly, RGD-targeted nanocarriers can transfer drugs into cancer cells by aiming the RGD peptide at overexpressed αVβ3 integrins, which enables active tumor targeting [75]. Fourthly, RGD-targeted nanocarriers can be internalized based on the receptor-mediated endocytosis, which is impossible with non-targeted nanocarriers [76].

**Figure 4:**
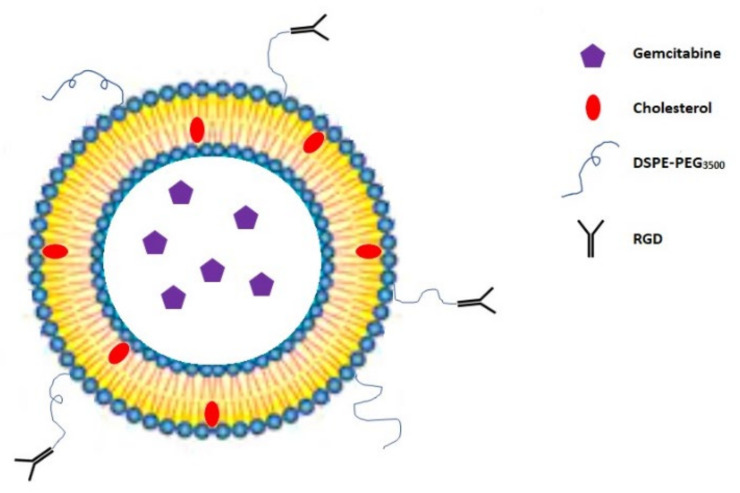
The structure of RGD-coated liposome with encapsulated gemcitabine. DSPE: distearoyl-sn-glycero-3-phosphoethanolamine [45].

Temming et al. reported that the conjugation of a (&RGDfK&) peptide on the—liposomes surface (Figure 4) significantly improves the intracellular gemcitabine uptake in the human ovarian cancer cell line SKOV-3. Additionally, this conjugate is relatively neutral towards human normal red blood cells (RBCs) [45]. Higher intracellular drug concentration caused by (&RGDfK&) loaded liposomes may affect crucial processes in cancer cells, such as the reduction of the mitochondrial membrane potential (DΨm) or the increase in the reactive oxygen species (ROS) level, inducing the apoptosis in cancer cells [77,78]. Unlike the necrosis, the apoptosis is a genetically-controlled process which does not entail inflammation in the human body, thus most of the chemotherapeutic drugs are designed to induce this process [79]. Tang et al. [80] showed that PEGylated liposomes (LPs) consisting of RGD tripeptide and gemcitabine upregulate the pro-apoptotic Bax protein, downregulate the anti-apoptotic Bcl-2 protein expression, and increase the apoptosis related protein caspase-3 expression in SKOV3 cell lines in comparison to the free drug and gemcitabine LPs without RGD peptide. Moreover, they determined that the RGD-Gem-LPs conjugate improves pharmacokinetic properties of gemcitabine, such as the biological half-life (t_1/2_), the bioavailability (AUC), and the mean residence time (MRT) after intravenous administration to rats. Cai et al. [46] confirmed that the RGD coating of Gem-LPs results in a lower toxicity and a much higher efficacy in MDA-MB-231 bearing mice compared with non-modified drug liposomes. This coated nanocarriers also exhibited a more beneficial entrapment efficiency (EE), physical stability, particle size, and shape than the liposomal gemcitabine. Yu et al. [47] showed that the (&RGD&) peptide conjugation on the surface of gemcitabine albumin nanoparticles (Gem-HSA-NPs) could be beneficial for pancreatic cancer patients. The in vitro results conducted on the BxPC-3 cell line confirmed that the (&RGD&) anchored nanoparticles can deliver gemcitabine to cells more efficiently than the non-targeting (&RGD&)-conjugated nanoparticles. After conjugation with the (&RGD&) peptide, the cellular uptake of (&RGD&)-HSA-NPs increased 3-fold in comparison with that of HSA-NPs. These results confirmed that the coating of the particle surface with (&RGD&) significantly enhances the uptake of nanoparticles by BxPC-3 cells. Moreover, when comparing with the free gemcitabine, 4-*N*-myristoyl-gemcitabine, and Gem-HSA-NPs, (&RGD&)-Gem-HSA-NPs exhibited a significantly higher apoptosis rate. This result was consistent with the cytotoxicity study and indicates that (&RGD&)-Gem-HSA-NPs induced stronger early and late apoptosis. This effect was caused by major gemcitabine intracellular uptake resulting from the active targeting by the (&RGD&) peptide giving higher cytotoxicity than the other compounds.

#### Co-Delivery of Gemcitabine and Paclitaxel in (&RGD&)-Modified Nanoparticles

The overexpression of αVβ3 integrin is correlated with the bone metastasis in breast cancer [81] and induces increased tumor growth and osteopontin response disorders [82]. The expression of αvβ3 participates in the regulation of the breast cancer cell reaction to chemotherapy and serves as a marker of chemosensitivity [83]. This integrin is upregulated in the cells treated with the microtubule interfering agents and the upregulation is not observed in the cell lines resistant to these drugs. However, the overexpression of the β3 subunit increases cell resistance to paclitaxel (Ptx) [65]. The paclitaxel/gemcitabine combination chemotherapy is one of the preferred treatments for metastatic breast cancer patients. The combination chemotherapy of paclitaxel and gemcitabine has survival benefits and a tolerable toxicity profile [84]. This combination strategy reduces the risk of insufferable side effects and maximizes the therapeutic effect, even at a lower dose [85,86,87]. Nevertheless, co-delivery of gemcitabine and paclitaxel without carriers is limited by various problems. These include, e.g., short t_1/2_, instability, and low cellular permeability of gemcitabine and the hydrophobic character of paclitaxel. To solve this problem, Zhang et al. [48] encapsulated gemcitabine monophosphate and paclitaxel in the (&RGDfC&)-modified nanoparticles. Studies conducted on MCF-7 tumor-bearing mice showed a prolonged elimination from the bloodstream and a higher maximum plasma concentration for paclitaxel and gemcitabine monophosphate (Gmp) in comparison with the free paclitaxel and gemcitabine (*p* < 0.05). Moreover, tumors treated with modified nanocarriers exhibit a decrease in the number of mitotic figures, more basophilic and uniform nuclei, and the reduction in the expression of Bcl-2 and Bcl-xL proteins. These studies also demonstrated that there is no significant difference between Ptx/Gmp-NPs and the untreated group in the levels of white blood cells, red blood cells, hematocrit, and hemoglobin. Despite the inevitable presence of the nanocarriers accumulated in the lungs or liver, their amount was significantly lower when comparing it with the tumor tissue. With the help of nanoparticles containing (&RGD&) as a targeting ligand, most of the nanoparticles are taken up by tumor cells.

### 3.3. Multifunctional Gemcitabine TPE-Gem-RGD Conjugate

**Figure 5:**
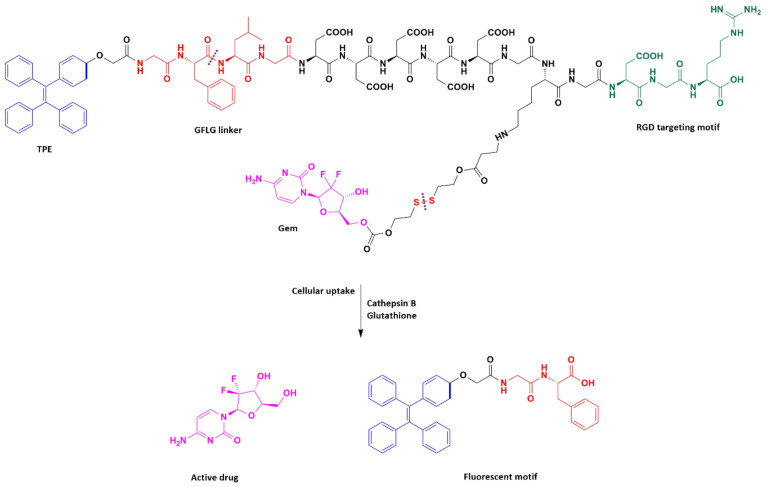
The structure of TPE-Gem-RGD for cathepsin B-responsive fluorescent product and reduction-responsive drug release. After cleavage of GFLG peptide by cathepsin B, intracellular fluorescence light up could be achieved. The conjugated anticancer drug- gemcitabine- is expected to be released upon the trigger of high intracellular glutathione concentration.

Han et al. [49] designed and synthesized an innovative multifunctional gemcitabine prodrug TPE-Gem-RGD which can be used for the targeted intracellular light-up imaging and the selective release of gemcitabine under the reductive environment inside cells. This conjugate contains: (1) Gemcitabine as a chemotherapeutic drug, (2) tetraphenylene (TPE) for the cell imaging, (3) GFLG (glycyl-l-phenyloalanyl-l-lecylglycine) linker sensitive to cathepsin B [88], (4) five residues of Asp to improve solubility of the conjugate, and (5) RGD tripeptide as a targeting group (Figure 5). Their studies proved that the RGD-targeted TPE-Gem-RGD conjugate is more effective in the inhibition of the BxPC-3 pancreatic cancer cells proliferation in comparison with the gemcitabine and the non-targeted TPE-Gem-RDG prodrug. This suggests that active targeting is a meaningful aspect of the anticancer drug design process. Moreover, the TPE-Gem-RGD prodrug is internalized by cells through RGD-mediated endocytosis, which makes this conjugate more effective in the suppression of BxPC-3 cells than TPE-Gem-RDG.

### 3.4. RGDV-Gemcitabine Conjugate

In order to prolong the half-life, overcome the drug resistance, and eliminate the bone marrow toxicity of gemcitabine, Liu et al. [50] designed and synthesized the RGDV-gemcitabine conjugate (Figure 3B). A lot of assays were carried out, including in vitro half-life assay, in vitro drug resistance assay, in vivo anti-tumor assay, in vivo kidney toxicity assay, in vivo liver toxicity assay, and in vivo marrow toxicity assay. Results, performed on a S180 tumor-bearing mouse model, indicated a 100-fold lower minimal effective dose, 10-fold higher anti-tumor activity, and l7-fold longer half-life (in mouse plasma) of the RGDV-gemcitabine conjugate in comparison with gemcitabine alone. Moreover, based on FT(+)-MS spectrum analysis, RGDV-gemcitabine does not enter the liver, kidney, and marrow of the treated tumor-bearing mice. This means that the conjugate showed no kidney toxicity, no liver toxicity, and no marrow toxicity. To evaluate the drug resistance, the dipyridamole (a potent nucleoside transporter inhibitor) was used against the proliferation of A549 cells. The results showed that dipyridamole elevates the IC_50_ of gemcitabine from 2.5 μM to 48.0 μM (19.2-fold increase) but has little influence on the IC_50_ of RGDV-gemcitabine (elevation from 2.5 μM to 4.7 μM, only 1.9-fold increase). Thus, the modification of Arg-Gly-Asp-Val successively reverses dipyridamole-induced drug resistance. There is no significant differences between IC_50_ values for gemcitabine and RGDV-gemcitabine inhibiting the proliferation of MCF-7, HCT-8, A549, 95D, and HepG2 cells. Therefore, the Arg-Gly-Asp-Val modification does not change the in vitro anti-tumor activity of gemcitabine. The results also showed that the RGDV-gemcitabine conjugate has nano-properties, and that an aqueous RGDV-gemcitabine is a nano-solution. This conjugate in ultrapure water, pH 6.7 can form small and uniform nano-particles. Over seven days, the size of nano-particles falls within a range of ~90–100 nm, the height of the nano particles is less than 18 nm, the diameter is less than 100 nm, and the nano-particles height in mouse serum is less than 8 nm. Such a nano-particle can specifically cross the cancer cell membrane and selectively enter the tumor tissue, thereby showing the tumor-targeting effect. The tumor-targeting action provides the high efficacy and low toxicity of RGDV-gemcitabine with a specific pharmacophore release. RGDV-gemcitabine in a nano-particle form can evade the macrophage uptake, enabling its delivery via the blood circulation in unmetabolized form, thereby prolonging the half-life and overcoming the drug resistance [50].

To summarize, these studies demonstrate that the specific targeting of the tumor angiogenesis-related αVβ3 integrin is possible using conjugates based on RGD-peptides. The majority of presented conjugates showed improved cytotoxicity specifically against cancer cell lines (BxPC-3, SKOV-3, MCF-7), remaining relatively inert towards non-target cells. Moreover, they exhibited enhanced pharmacokinetic properties of free gemcitabine, such as prolonged elimination from the bloodstream and a higher maximum plasma concentration. The recognized association of integrin receptors with tumor progression is a valuable basis for future preclinical and clinical studies, along with the development of targeted drug delivery systems. On the other hand, anticancer effects of presented conjugates with gemcitabine were marked in vitro on cell line-based models or in vivo on tumor-bearing mice, and the possible clinical application on humans should be considered carefully.

## 4. Conclusions

Drug targeting is crucial for effective cancer chemotherapy. Targeted delivery enhances chemotherapeutic effect and spares normal tissues from the toxic side effects of these powerful drugs. Up to now, many approaches have been made to improve the therapeutic index of gemcitabine. Peptide conjugates provide a valuable alternative to anti-cancer drugs used so far. A proper use of biological mechanisms of constituent peptide action can result in effective therapy for many diseases. Two groups of peptides were used to obtain gemcitabine conjugates—cell penetrating peptides and RGD peptides. Published results confirmed that peptides of both groups could be successfully applied to design new, efficient, and specific anti-cancer therapeutic agents, also in conjunction with the nanocarriers, such as nanoparticles, liposomes, or micelles. Enhanced pharmacological activity was achieved when components were non-covalently and covalently bond through the drug’s functional groups. The latter means that these groups could be subjected to the successful modification which usually is not the case for commercially available drugs. This again proves that gemcitabine is a very attractive leading structure to design gemcitabine conjugates with a potential to become new therapeutic tools for cancer therapy.

In conclusion, the use of gemcitabine peptide-based conjugates to treat cancer is a relatively new field and there are still many areas that need to be explored. However, these conjugates show great promise in the field of anticancer therapy because of their many benefits including no hematological or other toxicities, higher stability, and tissue specificity. One can expect that the successful transition from the laboratory to the clinic is only a matter of time and while the shift from the laboratory to the clinic is time consuming, and recent progress should promote this translation.

## Figures and Tables

**Table 1:** Gemcitabine-peptide conjugates designed for the anticancer therapy.

Conjugate	Type of Linker	Target (Cell Line)	Results	Ref.
*Gemcitabine Conjugate with Cell-penetrating Peptides (CPPs)*				
**Gem-Cys-pVec, Gem-Cys-Pen**	Disulfide bridge	Three human cancer cell lines: MKN-28 (human gastric cancer), Caco-2 (heterogeneous human epithelial colorectal adenocarcinoma), and HT-29 (human colon adenocarcinoma)	Longer half-life: 9.6 days for Gem-Cys-Pen) and 42 h for Gem-Cys-pVec MKN-28, Caco-2 and HT-29 IC_50_ < 50 µM *, gemcitabine > 100 µM	[41]
**dFdC-C2-CPP6-1,** **dFdC-C2-CPP6-2,** **dFdC-C2-CPP6-3**	Succinyl spacer	Human pancreatic adenocarcinoma (BxPC-3), human breast adenocarcinoma (MCF-7), and human prostate adenocarcinoma (PC-3) cancer cell lines	IC_50_:15 ± 0.6 nM for dFdC-C2-CPP6-1; 14 ± 0.4 nM for dFdC-C2-CPP6-3 and 74 ± 6.1 nM for gemcitabine alone in the PC-3 cell line	[32]
**[RW]3-Gem[RW]4-Gem** **[RW]5-Gem** **[RW]6-Gem** **R5W3R4-Gem**	Succinyl spacer	A549 cell line	Conjugates display increased toxicity compared with the free drug	[42]
*Gemcitabine Conjugate with Receptor-Binding Peptides*				
**Gemcitabine-succinate-GnRH (GSG)**	Ester linkages (four or five carbons) glutaric or succinyl spacer	Prostate cancer (CaP) cell lines (DU145 and PC3) Xenograft animal model Bone marrow cells derived from male C57BL/6 mice	IC_50_: 308 ± 170 nM for GSG and 231 ± 34 nM for gemcitabine GSG efficacy was achieved with a significantly lower dose when compared with gemcitabine (approximately 25 times) IC_90_: 41.4 ± 13.3 nM for GSG vs. 20.9 ± 8.5 nM for gemcitabine; IC_50_: 24.3 ± 6.4 nM vs. 12.1 ± 6.7 nM, respectively	[42] [43] [18]
Internalized-RGD ** (iRGD) peptide with gemcitabine	None, the mixture of gemcitabine and iRGD	Five mouse pancreatic cancer cell xenograft models: AsPC-1, BxPC-3, Capan-1, MIA PaCa-2, SUIT-2	iRGD peptide demonstrated a substantial booster accumulation effect of drugs in the mouse pancreatic cancer models with high NRP1 expression	[44]
**RGD peptide-gemcitabine-loaded nanocarriers**	Drug encapsulation	Human ovarian cancer cell line SKOV-3, human breast adenocarcinoma cell line MDA-MB-231 and MCF-7, and human pancreatic cancer cell line BxPC-3	IC_50_: 0.1 μg/mL for cRGD-Gem-HSA-NP, 0.28 μg/mL for gemcitabine, 0.38 μg/mL for Gem-C14, and 0.42 μg/mL for Gem-HSA-NP	[45,46,47,48]
**Multifunctional gemcitabine prodrug TPE-Gem-RGD**	GFLG *** tetrapeptide, disulfide bond	Human pancreatic cancer cell line BxPC-3	RGD-targeted TPE-Gem-RGD prodrug was inhibiting the proliferation of pancreatic cancer cells more efficiently	[49]
**RGDV-gemcitabine conjugate**	Directly connected via amide bond	Cell lines: MCF-7, HCT-8, A549, 95D, and HepG2	The IC_50_ values of gemcitabine and RGDV-gemcitabine show no significant difference; half-life of RGDV-Gem is 17-fold higher than for gemcitabine alone, no kidney toxicity, no liver toxicity, no marrow toxicity, and no drug resistance. The minimal effective dose and activity of RGDV-gemcitabine are 100-fold lower and 10-fold higher than that of gemcitabine, respectively	[50]

* with the only exception of Gem-Cys-pVec conjugate IC_50_ > 100 µM and Gem-Cys-Pen IC_50_ = 67.13 ± 2.92 µM against Caco-2 cell line. ** RGD-l-arginylglycyl-L-aspartic acid (Arg-Gly-Asp). *** GFLG- glycyl-L-phenyloalanyl-L-leucylglycine (Gly-Phe-Leu-Gly).

## Data Availability

No new data were created or analyzed in this study. Data sharing is not applicable to this article.
